# Upper Airway Changes and OSAS Risk in Patients after Mandibular Setback Surgery to Treat III Class Skeletal Malocclusion

**DOI:** 10.3390/jpm13071105

**Published:** 2023-07-07

**Authors:** Paolo Ronchi, Sabina Saccomanno, Barbara Disconzi, Stefano Saran, Andrea Carganico, Salvatore Bocchieri, Rodolfo Francesco Mastrapasqua, Luca Fiorillo, Sergio Sambataro, Marco Cicciù, Luca Levrini

**Affiliations:** 1 Department of Human Sciences, Innovation and Territory, School of Dentistry, Postgraduate of Orthodontics, University of Insubria, 21100 Varese, Italy; paoloronchi22@gmail.com (P.R.); barbaradisconzi97@gmail.com (B.D.); ssaran@studenti.uninsubria.it (S.S.); acarganico@studenti.uninsubria.it (A.C.); luca.levrini@uninsubria.it (L.L.); 2 Orthodontic Residency, Department of Life, Health and Environmental Sciences, University of L’Aquila, 67100 L’Aquila, Italy; sabinasaccomanno@hotmail.it; 3 ENT Department, Rivoli Hospital, ASL TO 3, 10098 Torino, Italy; rfmastrapasqua@aslto3.piemonte.it; 4 Department of Public Health Dentistry, Dr. D.Y. Patil Dental College and Hospital, Dr. D.Y. Patil Vidyapeeth, Pimpri, Pune 411018, India; lfiorillo@unime.it; 5 Department of Biomedical and Dental Sciences, Morphological and Functional Images, University of Messina, 98100 Messina, Italy; ssambataro@centrodiortodonzia.it; 6 Multidisciplinary Department of Medical-Surgical and Odontostomatological Specialties, University of Campania “Luigi Vanvitelli”, 80121 Naples, Italy; 7 Department of Biomedical and Surgical Sciences, University of Catania, 95124 Catania, Italy; mcicciu@unime.it

**Keywords:** OSAS, mandibular setback surgery, class III malocclusion, posterior airway space

## Abstract

Introduction: Mandibular setback surgery (MSS) is one of the treatment options to resolve mandibular prognathism in patients suffering from skeletal class III malocclusion, which cannot be treated with simple orthodontic treatment. The mandibular setback surgical operation can involve changes in the pharyngeal morphology, resulting in a narrowing of the posterior airway space (PAS). This aspect is associated with an increase in airflow resistance, which increases the risk of developing snoring or obstructive sleep apnea syndrome (OSAS). The aim of this study is to evaluate the medium- and long-term effects of mandibular setback surgery on the upper airways and its possible association with OSAS in patients suffering from class III skeletal malocclusion. Material and methods: A total of 12 patients (5 males and 7 females) were enrolled in this study. The statistical tests highlighted a significant change in the PAS and BMI values in relation to T0, before surgery (PAS: 12.7 SD: 1.2; BMI: 21.7 SD: 1.2), and T1, after surgery (PAS: 10.3 SD: 0.6, *p* < 0.01; BMI: 23.8 SD: 1.2, *p* < 0.05). Sample size was calculated to detect an effect size of 0.9, with statistical power set at 0.8 and the significance level set at 0.05. Results: No statistically significant correlation was found between the extent of mandibular setback, PAS and BMI change. Conclusion: This study confirms the effects of mandibular setback surgery on the upper airways, reporting a statistically significant PAS reduction in the medium- and long-term follow-up. On the other hand, no direct correlation was identified with OSAS risk, at least for the small mandibular setback (<8 mm), despite the statistically significant increase in BMI.

## 1. Introduction

Mandibular setback surgery (MSS) is one of the therapeutic options for the correction of mandibular prognathism in subjects suffering from severe class III skeletal malocclusion, which is untreatable with orthodontic treatment alone [1].

The indication for isolated mandibular setback surgery is limited to about 10% of cases that require surgical correction of this dentoskeletal dysmorphia [2]. Bimaxillary surgery is generally preferred [3]. This trend is explained by the low incidence rate of isolated mandibular prognathism, compared to cases in which mandibular prognathism is combined with retrognathism of the maxilla. In addition, bimaxillary surgery results in a better aesthetic effect and a reduced impact on the morphology of the upper airways [4].

The effect of orthognathic surgery on the upper airways has gained a lot of interest in recent decades [5].

The repositioning of the jaws, required by orthognathic surgery, causes an alteration in the skeletal facial anatomy and inevitably affects the relationship between hard and soft tissues. This involves changes in the position and tension of the anatomical structures associated with the jaw, such as the soft palate, tongue, hyoid bone, and orofacial muscles.

Depending on the direction and extent of the skeletal movements, an alteration in the volume of the nasal and oral cavities and in the size of the PAS (posterior airway space) can occur [5,6].

The mandibular setback surgical operation involving the aesthetic and functional correction of the mandibular prognathism, may also involve changes in the pharyngeal morphology, resulting in a narrowing of the PAS [7].

Research in this area has shown that the morphology and size of the upper airways are closely correlated with obstructive sleep breathing disorders. The narrowing of the PAS is associated with an increase in airflow resistance, increasing the risk of developing phenomena such as snoring and obstructive sleep apnea syndrome (OSAS) [4].

OSAS is a common chronic respiratory disorder characterized by repetitive collapses of the upper airways, which causes sleep fragmentation, oxygen desaturation, and excessive daytime sleepiness. This disorder is associated with decreased quality of life, increased all-cause mortality, and significant medical comorbidities, such as cardiovascular disease, cerebrovascular events, diabetes, and cognitive impairment.

A potential narrowing of the PAS following mandibular setback surgery could therefore be a predisposing factor for the onset or aggravation of OSAS [8].

The gold standard for the diagnosis of OSAS is polysomnography, however its use in epidemiological studies, especially in large populations, is limited due to the cost, human resources and logistics required.

A few cases in which OSAS occurred after a mandibular setback surgery have been reported in the literature [9]. However, a recent systemic review of the literature concluded that there is no clear association between mandibular setback surgery and the onset of OSAS [8]. Therefore, the potential role of this restriction in the development of OSAS remains a hotly debated topic [10].

The aim of this study is to evaluate the medium- and long-term effects of mandibular setback surgery on the upper airways and the possible association with OSAS, in patients suffering from class III skeletal malocclusion.

The null hypothesis assumes that mandibular setback surgery could influence the upper airways and increase the risk of OSAS.

The evaluation of the upper airways can be performed through cephalometric analysis in the sagittal plane and through the analysis of the score obtained in the Berlin questionnaire, which is a self-assessment test that identifies the level of risk of OSAS onset. More accessible screening methods to detect patients at high risk of OSAS and to refer them to a subsequent polysomnographic evaluation could be beneficial. Various approaches can be used in this regard, including the Berlin, Epworth, STOP and STOP-BANG questionnaires.

The validity of the Belin questionnaire has been examined in a wide variety of populations, but the sensitivity and specificity vary from study to study. However, it is commonly used in epidemiological and clinical research [11].

## 2. Materials and Methods

This work is a retrospective study conducted on patients who had undergone mandibular setback surgery at the ASST Lariana, Sant’Anna Hospital in Como.

### 2.1. Population and Study Design

A total of 12 patients who had undergone mandibular setback surgery for the correction of a severe class III skeletal malocclusion were enrolled in this study. Sample size was calculated to detect an effect size of 0.9, with statistical power set at 0.8 and significance level set at 0.05.

Patients were recruited from January 2021 to January 2022, between 2 and 40 years after surgery. The mean follow-up was 27.6 years. All the patients underwent fixed orthodontic treatment before the surgery.

As already highlighted above, the relatively small number of cases is due to the fact that there are few patients with class III skeletal malocclusion in which there is an indication of an isolated mandibular setback surgery.

All patients were informed and gave their consent to the processing of data.

The following data were collected for each patient:

- Sociodemographic, anthropometric data:
  ∘ Gender: 5 males, 7 females;
  ∘ Age at the time of surgery: between 19 and 40 (mean 24.6) years;
  ∘ Preoperative BMI: variable between 17 and 32 (mean 21.7) kg/m^2^.
- Operating parameters relating to the mandibular retraction surgery:
  ∘ The surgical approach used: the surgery was performed, in all patients, by means of bilateral sagittal osteotomy of the mandibular angle according to Obwegeser-Dal Pont, modified according to Gotte.
- Preoperative radiographic documentation:
  ∘ Lateral teleradiography of the head.

After a variable follow-up that ranged from 2 to 40 years, with an average follow-up of 27.6 years, the patients underwent:

- Cephalometric analysis based on pre- and postoperative lateral teleradiography of the head to assess the extent of the mandibular setbacks and morphological changes in the upper airways.

Berlin questionnaire: a self-assessment that identifies the degree of risk of OSAS onset.

### 2.2. Evaluation Tools

#### 2.2.1. Cephalometric Analysis

All the patients involved in the study, after a variable period of time since the surgery, underwent a teleradiograph of the head in order to compare it to the preoperative one.

Once the radiographic image was obtained, the cephalometric analysis was performed using the cephalometric tracing proposed by Caprioglio et al. [12]. Through the identification of points, angles, and anatomical reference planes, cephalometry enables the following to be examined in detail: the craniofacial structure, the sagittal and vertical skeletal relationship, the dental relationship, the dental–basal relationship, the profile of the soft tissue, and the upper airway patency.

The following cephalometric parameters were analyzed in this study, in both the pre-and postoperative teleradiography of the head:

- The PAS, the distance between the posterior pharyngeal wall and the back of the tongue.
- The Basion–Pogonion distance: parameter used to evaluate the extent of mandibular setback, as already described by Schulten et al. [11]. Although it is not easy to identify, we used the Basion point, rather than the Porion point, as the condyle, following mandibular osteotomies, can undergo modifications that are also quite significant both in terms of the position and the shape.

Intraclass correlation coefficient (ICC) was used to evaluate reliability, resulting in a high degree of reliability (ICC > 0.91 for all measurements).

#### 2.2.2. Berlin Questionnaire

For the risk assessment of OSAS following mandibular setback surgery, we used the Berlin questionnaire.

This questionnaire consists of ten questions, in addition to general information relating to weight and height, arranged in three categories relating to the risk of OSAS:

- Snoring and breathing cessation (five questions);
- Symptoms of excessive daytime sleepiness (four questions);
- Hypertension (one question) and BMI.

The BMI, calculated using the formula $\frac{Weight\;\left(Kg\right)\;\times \;1000}{{High\;\left(cm\right)}^{2}}$, is incorporated in this OSAS risk assessment questionnaire since it has been shown that a high value, corresponding to a BMI > 28, results in an 8-to-10-time increased risk of OSAS, and that 10% weight gain above the normal limit leads to a 6-time increase in OSAS risk [13].

Obesity is one of the most important OSAS risk factors since the loss of muscle strength in the upper respiratory tract due to the accumulation of fat on the muscles reduces the diameter of the airways. Obesity reduces total respiratory compliance, decreasing both chest and lung wall compliance. Consequently, while the residual functional capacity, vital capacity, and total lung capacity decrease, the resistance of the airways increases, predisposing the patient to the onset of obstructive sleep disorders [13].

Based on their responses and the overall rating in the categories of Berlin Questionnaire, patients can be classified at high or low risk of OSAS.

## 3. Results

The extent of mandibular setback, assessed through cephalometry by evaluating the distance between Pogonion and Basion, was found to be between 4 and 8 (mean 5.7) mm.

PAS preoperative values ranged from 7 to 19 (mean 12.7) mm, while postoperative values ranged from 7 to 14 (mean 10.3) mm.

The cephalometric evaluation showed that isolated mandibular setback surgery led to a decrease in PAS values in 9 of the 12 selected patients.

This reduction was more significant in patients with high preoperative PAS, while in patients with lower PAS values (patients n° 6, 7, and 9), no changes in the PAS were found.

Patient n° 1 reported a PAS reduction from 15 to 12 mm after mandibular setback surgery of 5 mm, confirming that PAS reduction is more evident in patients with a higher initial PAS.

The Berlin questionnaire revealed a low risk of OSAS in almost all patients, despite the increase in BMI in some patients. The presence of OSAS was only found in one patient (n° 9) at a 27-year follow-up. This patient showed a particular clinical history, characterized by a notable weight gain (20 kg) during a pregnancy at the age of 35, which then regressed very slowly. Already in the last months of pregnancy, a progressive and significant increase in snoring had begun with the subsequent appearance of the first symptoms of moderate OSAS at the age of 50 (AHI = 25.4). The apneas in this patient were mainly positional, and she is currently being treated with an oral appliance, as shown in Table 1.

### Statistic Analysis

For the PAS and BMI variables, the Shapiro–Wilk normality test was performed, and the mean was then compared using the paired-sample Student *t*-test (Table 2).

**Figure 1:**
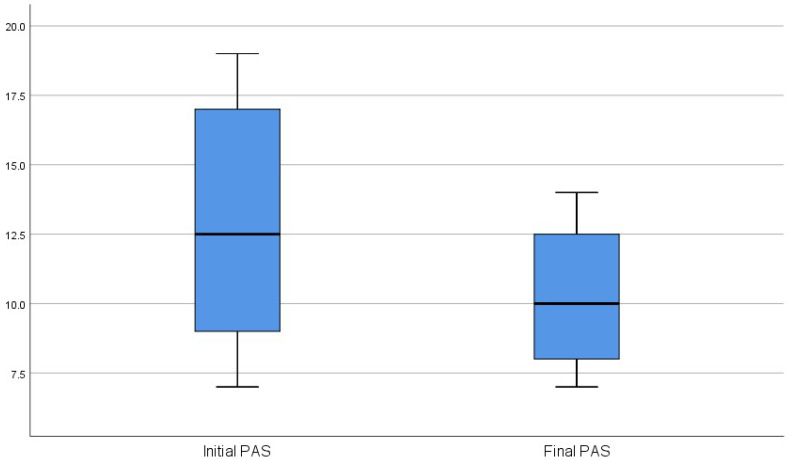
Box plot to compare the data distribution of the initial and final PAS score.

**Figure 2:**
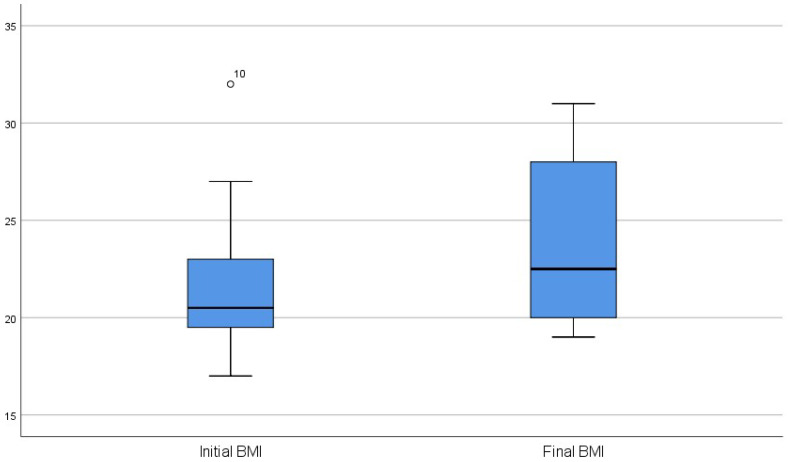
Box plot to compare the data distribution of the initial and final BMI score.

The statistical tests highlighted a significant change in the PAS and BMI values in relation to T0 (before surgery) and T1 (after surgery). No statistically significant correlation was found between the extent of mandibular setback, PAS, and BMI change, as shown in Figure 1 and Figure 2.

## 4. Discussion

PAS can be influenced by several factors, such as head posture, tongue position, age, body mass index (BMI), as well as surgical or orthodontic displacement of the mandibular position [14].

The present study showed that the surgical treatment of isolated mandibular setback in patients with class III skeletal malocclusion can lead to short-, medium- and long-term PAS reduction. PAS preoperative values ranged from 7 mm to 19 mm (mean 12.7 mm, SD 1.2), while postoperative values ranged from 7 mm to 14 mm (mean 10.3 mm, SD 0.6).

The most significant changes in PAS emerged in patients with higher preoperative PAS. Conversely, in cases where the initial PAS was at the lower threshold limit, there was either no or a non-significant postoperative size reduction.

Furthermore, the post-surgical PAS modifications and the risk of OSAS onset appeared to be independent. Only one of the patients examined showed OSAS onset after a 27-year follow-up after surgery. In this case, the postoperative PAS showed no changes after a mandibular setback of 6 mm. In addition, this patient underwent a significant increase in BMI, which slowly regressed over the years. There therefore seems to be no real cause–effect relation between MSS and OSAS, but rather a sporadic event due to environmental risk factors.

Many studies have investigated the effects of skeletal displacements caused by orthognathic surgery on the upper airways, and some have gone further and studied their relationship with obstructive sleep breathing disorders.

We carried out a literature review of studies that radiographically assessed the impact of mandibular setback surgery on the upper airways. This review was designed to investigate the effects of a specific maxillofacial surgical procedure, as the combination of additional surgical procedures would have made it more difficult to predict changes in the soft tissue and airways [15].

For this reason, all studies were excluded that involved surgical interventions other than mandibular setback surgery with the SSRO (sagittal split ramus osteotomy) technique for the correction of class III skeletal malocclusions.

Overall, we analyzed 12 publications in detail. Kim et al. (2021) [16] studied a sample of 28 patients (16M, 12F), with 22.9 as the mean age. They found a decrease in airway size immediately after MSS, followed by a partial recovery during short-term follow-up (T2), which was maintained until long-term follow-up (T3). Irani et al. (2018) [17] analyzed a sample of 28 patients (17M, 11F), with 23.88 as the mean age. They reported that oropharyngeal, hypopharyngeal, and total volumes were reduced up to 6 months after MSS, after which they stabilized or partially recovered. Unlike other studies, there was no significant correlation between the reduction in PAS and the extent of mandibular displacement. Jeong et al. (2018) [18] studied a sample of 18 patients (10M, 8F). They found that there was a significant reduction in the transverse and anteroposterior section of the airways over a one-year period after MSS, and the largest decrease was observed in the oropharynx area. Shah et al. (2016) [19] included 29 patients (18M, 11F) in their study, with 23.67 as the mean age. They reported that the volume of the pharyngeal airways decreased and that the mean negative pressure increased, resulting in greater patient effort to maintain a constant pharyngeal airflow. Cho et al. (2014) [20] enrolled 13 patients (7M, 6F) with a mean age of 22.4. They reported a significant reduction in the size of the nasopharynx and oropharynx within two months after MSS, after which dimensional stability or partial recovery was recorded. They also reported a reduction in the hypopharynx that continued for more than six months post-surgery. In a sample of 50 patients (21M, 29F), with a mean age of 24.1, Choi et al. (2010) [21] found that PAS reduced within two months after MSS, after which the reduction was attenuated, resulting in a gradual and partial recovery in the six months after surgery. Marsan et al. (2008) [22] reported a significant reduction in the size of the pharyngeal airways for up to one year after MSS in a sample of 25 female patients, with 25.4 as mean age. Similarly, Muto et al. (2008) [23] found that retropalatal and retrolingual airways narrowed significantly one year after surgery in a sample of 49 female patients with a mean age of 24.5. Sato et al. (2005) [24], enrolled 30 patients (10M, 20F) in their study, with an overall age range of 18–37 years of patients undergoing MSS. They found that the space of the pharyngeal airways remained constant in the short term (T2), while in the long term (T3), there was a significant reduction in the posterior lingual airways. Eggensperger et al. (2005) [25], analyzed a sample of 12 patients (9M, 3F) with 28 as the mean age. They reported that after the initial decrease following MSS, the dimensions of the lower pharynx airways remained virtually unchanged, while the airway size of the upper and middle pharynx continued to decrease in the postoperative period. Saitoh K et al. (2004) [26] studied a sample of 10 female patients who underwent MSS with a mean age of 23.3. They concluded that from T1 to T2, the pharyngeal airways narrowed significantly, however the reduction had subsided and partially recovered in the long-term follow-up (T3). Considering 14 patients between 15 and 35 years of age, Tselnik et al. (2000) [27] found an immediate postoperative increase in PAS. On the other hand in the long-term follow-up, they found a significant reduction in both the PAS area and the anteroposterior dimension of the pharyngeal airways.

In this review, the follow-up duration ranged from 6 months to 12 years, at the end of which, in all cases, a reduction in the PAS emerged compared to the preoperative conditions.

Some of the changes that can occur after mandibular setback surgery are similar to the structural characteristics of patients with OSAS, including a reduction in the PAS and the inferior displacement of the hyoid bone [16]. Therefore, mandibular setback surgery could be one of the predisposing factors for the onset of sleep obstructive respiratory disorders, such as snoring and OSAS [9].

We thus analyzed further studies evaluating the effects of mandibular setback surgery on respiratory function by performing an instrumental examination to diagnose the possible development of OSAS.

Overall, we analyzed five publications that evaluated PAS changes and respiratory parameters after mandibular setback surgery with the SSRO technique. Patient groups undergoing bimaxillary surgery were excluded from the following studies.

Uesugi et al. (2014) [17] analyzed 22 patients who underwent respiratory monitoring during sleep after MSS. This test revealed no change in AHI in all patients except for one., The AHI of this latter patient increased from 14.9 events/hour to 19 events/hour after surgery, which is indicative of moderate OSAS, despite showing no significant change in pharyngeal airways morphology. The patient also presented first degree obesity, advanced age (54 years), and also underwent a relatively large mandibular setback (10.1 mm).

Kobayashi et al. (2013) [18] studied the oximetric parameters immediately after MSS in 21 patients and found a significant worsening compared to the preoperative patients, followed by a gradual improvement. There was no evidence of sleep breathing disorders six months after surgery because most patients adapted to the new conditions to ensure proper respiratory function during sleep.

Hasebe et al. (2011) [19] analyzed the respiratory parameters of 11 patients after MSS. They found that AHI did not change significantly, except for one patient who reported the onset of mild OSAS after MSS, even without a significant change in PAS. This 22-year-old male had no OSAS or obesity before surgery (preoperative AHI 4.4 events/h, BMI 20.6 kg/m^2^ before surgery and 19.8 kg/m^2^ 6 months after surgery). However, he did have a considerable mandibular setback (13.7 mm).

Kitagawara et al. (2008) [20] analyzed 17 patients in whom SpO2 decreased during sleep immediately after MSS, but improved in the first month following surgery. No evident signs of OSAS were recorded.

Yamada et al. (2008) [21] found no significant difference between pre and postoperative AHI or SpO2 values in all 11 patients involved in their study. The same conclusion was reached by Hochban et al. (1996) [22], who found no evidence of postoperative OSAS through respiratory monitoring during sleep in all 16 patients enrolled.

Other studies have investigated the cephalometric changes in patients undergoing anterior maxillary segmental distraction observing a decrease in nasopharyngeal area while compared to traditional approaches [23].

Almost all the studies investigated morphological changes of the airway space by two-dimensional teleradiography, except for one study, in which the chosen technique was three-dimensional CBCT.

Studies by Uesugi et al. [17], Kobayashi et al. [18], Yamada et al. [21], and Hochban et al. [22] showed a significant PAS decrease after a minimum of six months after MSS associated with marked displacements of the hyoid bone. Conversely, Hasebe et al. 2011 [19] and Kitagawara et al. 2008 [20] found that the postoperative PAS remained unchanged.

All the studies in this review evaluated respiratory parameters to investigate the possible onset of OSAS. Different methods were used: the measurement of blood oxygen saturation with the oximeter, respiratory monitoring during sleep with portable devices and polysomnographic examination. Although polysomnography is the best method to diagnose sleep breathing disorders, the costs are high, and it requires more technical skills. Respiratory monitoring at night and pulse oximetry are more easily manageable and more accessible than polysomnography, and thus represent a valid alternative as a screening tool.

Of the 98 patients analyzed in this review, only two patients demonstrated changes in respiratory parameters after MSS. A total of [19] 96 patients reported no signs of respiratory parameters worsening after surgery. In these patients, any narrowing of the PAS did not lead to an increase in AHI, probably due to functional readjustments in the surrounding muscles or tissues.

Based on this review of the literature, no direct cause–effect relationship emerged between mandibular setback surgery and OSAS development. Moreover, OSAS onset appears to be influenced by other genetic or environmental factors. It seems that since MSS is not associated with other risk factors, it does not cause OSAS in the 6-month follow-up after surgery. However, a relatively short follow-up could be a limitation of these studies, as there may be risks even in the long term. Therefore, careful observation of respiratory spaces is important both in the acute phase and in the subsequent period [21].

In the acute phase, the risk of obstruction could be caused by the initial narrowing of the PAS and post-surgical inflammatory phenomena, such as swelling, bleeding and exudation. On the other hand, in the long term, although almost all subjects are able to adapt to the new breathing spaces, there may be a risk of developing OSAS, especially in the presence of other risk factors, such as obesity, potential sleep breathing disorders, and a large amount of mandibular setback.

### Limitation and Fi-Index Tool

This manuscript has been checked with the fi-index tool and obtained a score of 0.13 for the first author only on 19 May 2023 according to SCOPUS^®^. The fi-index tool aims to ensure the quality of the reference list and limit any auto-citations. This study presents some potential limitations, such as the small sample size and the heterogeneity of subjects. In addition, the evaluation of OSAS through questionnaires is not as reliable as a clinical examination.

## 5. Conclusions

In agreement with the literature, our study confirmed the effects of mandibular setback surgery on the upper airways, finding a statistically significant reduction in the PAS during medium- and long-term follow-up. This reduction was greater in patients with an initially high PAS in comparison to patients with an initial low PAS, but without any statistical significance.

On the other hand, no direct correlation was identified with OSAS risk, at least for a small mandibular setback (<8 mm).

This does not mean that the OSAS risk should not be considered in a mandible setback surgical planning of a class III patient, especially for quantitatively significant isolated mandibular setback in patients with other predisposing risk factors.

When deciding the treatment plan, it is therefore essential to consider the risk of potential airways compromise and the risk of obstructive sleep disorders. Maxillary advancement or another non-narrowing airway technique may be better options for patients with class III skeletal malocclusion with large anterior–posterior discrepancy.

## Figures and Tables

**Table 1:** Results obtained from the cephalometric analysis and from the Berlin questionnaire.

Patients	Gender	Age	Mandibular Setback (mm)	Follow-Up	Initial PAS (mm)	Final PAS (mm)	Initial BMI (kg/m^2^)	Final BMI (kg/m^2^)	Berlin Questionnaire
1	M	26y	5	4y	15	12	20	20	Negative
2	F	19y	6	27y	17	13	21	20	Negative
3	M	22y	8	5y	17	14	21	25	Negative
4	F	20y	8	40y	15	11	17	19	Negative
5	M	22y	7	25y	17	12	22	28	Negative
6	F	40y	6	2y	7	7	20	20	Negative
7	F	24y	6	23y	8	8	18	22	Negative
8	F	20y	5	3y	10	9	19	21	Negative
9	F	31y	6	27y	8	8	27	29	Positive
10	M	28y	4	27y	10	8	32	31	Negative
11	F	22y	4	30y	10	9	20	23	Negative
12	M	22y	7	23y	19	13	24	28	Negative

**Table 2:** Results of *t*-test for PAS and BMI.

	Initial	Final	
PAS	12.7 ± 1.2	10.3 ± 0.6	*p* < 0.01
BMI	21.7 ± 1.2	23.8 ± 1.2	*p* < 0.05

## Data Availability

Data is available upon request to corresponding authors.
